# Reliability and validity of patient-reported outcomes measurement information system^®^ (PROMIS^®^) measures of pain in adult males with hemophilia

**DOI:** 10.1186/s41687-026-01176-0

**Published:** 2026-08-19

**Authors:** Tyler W. Buckner, Dianne Thornhill, Bryce B. Reeve, Thomas A. Wilkinson, Sonia Nasr, Marilyn J. Manco-Johnson

**Affiliations:** 1 Hemophilia and Thrombosis Center, University of Colorado Anschutz Medical Campus, Aurora, USA; 2 Department of Population Health Sciences, Duke University School of Medicine, Durham, USA; 3 Gloval LLC, Broomfield, USA

**Keywords:** Computerized adaptive testing, Hemophilia A, Hemophilia B, Pain, Patient-reported outcomes, Quality of life

## Abstract

**Background:**

Inadequate pain assessment in persons with hemophilia (PwH) can lead to undertreatment of hemophilia-related pain. Effective pain management in PwH is limited by the lack of valid, reliable pain assessment tools that can be readily incorporated into clinical practice. The objectives of this study are to evaluate the reliability and validity of the Patient-Reported Outcomes Measurement Information System^®^ (PROMIS^®^) pain intensity, interference, and behavior measures in PwH.

**Methods:**

PROMIS pain measures were evaluated in a cross-sectional study that consecutively enrolled males ages ≥ 18 years with hemophilia A or B during routine clinical visits. Participants provided responses to static short forms (for the pain intensity domain) or to computerized adaptive tests (for pain interference and pain behavior domains) using electronic devices. Content validity was assessed through one-on-one interviews. Reliability (internal consistency and test-retest reliability), known group validity, and convergent validity were also evaluated.

**Results:**

Responses to PROMIS pain measures were collected from 150 participants. Content validity for the PROMIS pain interference item bank was good, with no coverage gaps identified in eight one-on-one interviews. Internal consistency for PROMIS pain measures was high (marginal reliability ranging between 0.87 and 0.91), and PROMIS pain measures showed adequate test/retest reliability over 24–72 h (intraclass correlation coefficients 0.85–0.95). We observed strong to very strong correlations between PROMIS measures and Brief Pain Inventory and Short Form-36 pain subscales (Pearson correlation coefficient magnitudes of 0.66–0.88), indicating convergent validity for these measurements.

**Conclusions:**

Results from this cross-sectional study support the reliability and validity of PROMIS pain intensity, interference, and behavior measures when assessing patient-reported pain in male PwH.

**Supplementary Information:**

The online version contains supplementary material available at https://doi.org/10.1186/s41687-026-01176-0.

## Background

Pain that accompanies bleeding episodes is a common complication in persons with hemophilia (PwH), and recurrent joint bleeding episodes in PwH can lead to chronic and painful arthropathy that interferes with work, school, sleep, and quality of life [1–3]. Differentiating between persistent pain due to ongoing arthropathy and concurrent acute pain from a joint hemorrhage at the same location can be difficult for PwH, and this lack of differentiation can potentially impact treatment strategies for bleeding episode management and pain control [4–6]. Suboptimal pain assessment by healthcare providers can lead to the undertreatment of pain, as pain that is underestimated in severity might not be treated as aggressively as needed, and pain that goes unrecognized will not be treated at all. Despite the centrality of pain in the lives of PwH, pain is often insufficiently addressed in these patients [3, 6–9].

Optimal pain management in PwH depends upon the availability of pain assessment tools that provide meaningful information to clinical staff while also being practical to implement. The National Institutes of Health (NIH)-supported Patient-Reported Outcomes Measurement Information System^®^ (PROMIS^®^) encompasses a broad set of patient-reported outcome (PRO) instruments for measuring numerous domains of physical, mental, and social health [10, 11]. PROMIS includes pain measures quantifying pain intensity, pain interference (impact of pain on physical, mental, and social activities), pain behavior (outward signs of avoiding or reducing pain), and pain quality (referring to various sensory and affective attributes of pain) [11]. PROMIS item banks were developed using item response theory (IRT) [12]. The scores were calibrated so the center of the distribution is representative of the average in the US population norms, which facilitates interpretation of acquired results [10]. PROMIS may be administered via static short forms or computerized adaptive testing (CAT). During CAT, particular items are drawn from the entire item bank and presented to participants based on prior answers, with the potential advantage of reducing respondent burden while maintaining measurement precision [13]. CAT requires electronic data capture hardware (e.g. computer or tablet), a stable internet connection with access to secure remote servers, and data collection software such as Research Electronic Data Capture (REDCap) [14], which can interface with the PROMIS Assessment Center to administer questionnaires. The immediate, automated scoring of PROMIS measures can provide real-time, interpretable data that are potentially well-suited to use for clinical care.

While studies of PROMIS pain interference and pain intensity measures in adults and children with hemophilia from the Netherlands have supported the validity and feasibility of these PROMIS measures in the Dutch hemophilia population [15–17], available data regarding pain assessment with PROMIS in other PwH populations remain limited. Further testing of PROMIS pain assessment measures in PwH is needed to determine whether these instruments can provide accurate and meaningful reflections of hemophilia-related pain in diverse clinical settings. In the current cross-sectional study, we explored the performance of PROMIS pain intensity, interference, and behavior measures in men from the United States with hemophilia A or B ages 18 and older using CAT and short form questionnaires. We assess (i) whether PROMIS pain measures are relevant to PwH (content validity); (ii) whether PROMIS pain scales can discern between mild, moderate, and severe hemophilia (known group validity); (iii) whether PROMIS pain measures are correlated with the Brief Pain Inventory (BPI) and the Medical Outcomes Study Short Form-36 (SF-36) bodily pain subscale instruments [18] (convergent validity); and (iv) whether PROMIS pain scores at baseline and 24–72 h later are consistent over time when the person’s pain level has not changed (test-retest reliability). We hypothesized that PROMIS pain intensity, pain interference, and pain behavior scores would be higher (worse) in individuals with severe hemophilia than in individuals with mild or moderate hemophilia. We also hypothesized that PROMIS pain domain scores would be strongly correlated with scores obtained from the BPI and SF-36 legacy instruments, and that PROMIS pain domain scores would show strong positive correlations with those pain domain scores measured 24–72 h after initial questionnaire administration. The goal of our study was to test the validity and reliability of PROMIS pain measures in a population with a broad range of hemophilia-related pain experiences. Our overarching hypothesis is that PROMIS pain assessment scores represent reliable and valid measures of pain in adult PwH, and that PROMIS pain questionnaires can be readily used in the clinic in real-time during hemophilia patient visits.

## Methods

Males aged 18 years and older with congenital hemophilia A or B of any severity (mild, 5%-40% of normal factor VIII or IX activity; moderate, 1%-5%; severe, < 1%) and having the ability to speak and read English or Spanish were eligible for enrollment. To minimize variability among enrolled participants, women were excluded from this study owing to markedly different disease manifestations between sexes in hemophilia A or B patients. All potentially eligible study participants attending consecutive, routine clinical visits (in person or via telehealth) at the University of Colorado Hemophilia and Thrombosis Center (HTC) were offered participation until the target study sample size was reached.

Demographic and clinical data (age, race and ethnicity, hemophilia severity, use of prophylactic clotting factor, medical history, and comorbid conditions) were collected from electronic medical records. PRO questionnaires were completed using an electronic device (computer, tablet, or smart phone) with a stable and secure internet connection, with research staff providing participants with technical assistance in operating electronic devices as needed. All questionnaire data were collected and managed using web-based REDCap tools hosted on secure servers at the University of Colorado. CAT instruments were administered utilizing REDCap’s secure interface with the PROMIS Assessment Center Application Programming Interface. Instruments used in this study were available in both English and Spanish.

Participants rated current pain levels, as well as worst and average pain experienced (within last 7 days), on a scale from 0 (‘had no pain’) to 5 (‘very severe’) using the PROMIS Pain Intensity Short Form 3a instrument (v1.0) [19]. Pain interference and pain behavior domains were assessed using CAT-based administration of the Pain Interference Bank v1.1 and Pain Behavior Bank v2.0, respectively [20, 21]. PROMIS domain measurements were represented by normalized T-scores with a mean of 50 and a standard deviation (SD) of 10, referenced to the general US population. Higher T-scores for PROMIS pain measures reflect more of the pain construct being measured. For CAT-based assessment, a minimum of 4 and a maximum of 12 items were administered to each participant (Supplementary Table 1). CATs were terminated when the standard error (SE) of the estimated T-score fell below 3.0 or changed by less than 0.1 on the T-score metric, or when the maximum number of items allowed to be administered was reached [20, 21]. Participants who responded “had no pain” for all PROMIS pain behavior items administered were excluded from subsequent pain behavior domain analyses, as the PROMIS Pain Behavior v2.0 instrument was designed to be administered only to patients who are experiencing pain [21, 22].

The BPI Short Form and the SF-36 bodily pain questionnaire were used to evaluate convergent validity of the PROMIS measures [5, 18, 23–25]. The BPI Short Form scores 11 static items (including 4 items comprising pain intensity and 7 items making up pain interference subscales) with a 7-day recall period. The mean of the item scores was used to generate BPI subscale scores. The SF-36 scores 2 static items (1 item each for pain intensity and pain interference domains; Supplementary Table 1). The standard 4-week recall timeframe of the SF-36 bodily pain subscale items was changed to a 7-day recall to align with the recall period of the other measures in the study. Additional SF-36 items beyond the two pain items were not administered. BPI items were rated from 0 to 10, with higher scores reflecting worse pain or greater pain-related interference. SF-36 subscale scores were transformed to a scale ranging from 0 to 100, with higher scores reflecting less intense pain or decreased pain-related interference. Floor or ceiling effects were considered significant for a given pain domain if more than 15% of participants provided responses yielding the lowest or highest possible scores, respectively [26].

To evaluate the content validity of the PROMIS pain interference item bank, participants were invited to take part in one-on-one interviews with a trained research staff member. The content validity of the PROMIS pain behavior item bank was not captured in this study. Content validity interviews were conducted either face-to-face or by telephone. Participants were asked to review each item in the PROMIS pain interference item bank to ascertain participant perceptions of item comprehensibility and relevance to hemophilia-related pain, as well as to identify any important aspects of their pain experience that were not covered by the PROMIS pain interference item bank. Any such aspects reported by at least 3 participants were recorded as potential coverage gaps, and any items identified as not applicable/not comprehensible by at least 3 participants were noted.

Internal consistency for the static PROMIS pain intensity measure was assessed using Cronbach’s alpha, while a measure of marginal reliability, computed as 1 – ((mean SE^2^)/Var(T-scores)) [27], was used to evaluate internal consistency for the CAT-based measures. PROMIS pain measures were readministered 24–72 h after the initial assessment to measure test-retest reliability. Retest assessments took place outside of the clinical setting and were administered through an interactive online interface using REDCap software. Participants also reported a global rating of change (GRC) in pain experienced at this follow-up timepoint relative to pain experienced at study entry. Only participants who reported no change from baseline pain in response to the GRC question were included in test-retest reliability analyses, which were performed using intraclass correlation coefficients (ICCs) that were obtained from a two-way mixed effects model of absolute agreement [28]. Convergent validity of each PROMIS measure was also evaluated by examining correlations between the BPI, SF-36 bodily pain subscales, and PROMIS measures using Pearson’s correlation coefficient, with coefficients > 0.80 reflecting very strong correlation and coefficients of > 0.60 to ≤ 0.80 reflecting strong correlation [29, 30]. Known group validity was evaluated by using analysis of variance (ANOVA) and independent samples t-tests to determine whether PROMIS pain intensity, pain interference, and pain behavior scores differ significantly based on hemophilia severity group (i.e., non-severe [mild or moderate] vs. severe, or mild vs. moderate vs. severe). The size of the study was selected to detect a 5-point difference separating the 2 or 3 known severity groups with 80% power at a significance level of 0.05. Statistical analysis was performed using SPSS version 31.

## Results

The study enrolled 158 males, with 150 participants (95%) completing the PROMIS, BPI, and SF-36 questionnaires. Eight participants were lost to follow-up after enrollment and did not complete questionnaires. Data collection took place from August 2019 to March 2023. Study participants needed minimal technical assistance in operating the electronic devices (computer, tablet, or smart phone) used for providing responses to questionnaires, indicating that these platforms were intuitive to use. While questionnaires were offered in both English and Spanish, none of the participants elected to use the Spanish language questionnaires. The median age of study participants who completed questionnaires was 37 years and ranged from 19 to 76 years (Table 1). Approximately half of participants had severe hemophilia A or B, and 11% had an active inhibitor or an inhibitor history. 60% of all participants and 97% of those with severe hemophilia were receiving routine prophylaxis treatment, and 30% (one-half of the participants using prophylaxis) were receiving emicizumab. Three participants had received gene therapy treatment and were not using clotting factor prophylaxis (Table 1). 57% of participants used medications for pain relief (with 21% treating with medications at least 5 days a week), and 63% used some form of non-pharmacological pain therapy (Table 2).

**Table 1:** Demographics and clinical characteristics of study participants who completed questionnaires

Participants (*N* = 150)	Summary
Age (y)	
Mean (SD)	40 (15)
Median	37
Minimum/maximum	19/76
Hemophilia type and severity, N (%)	
Hemophilia A	115 (76.7)
mild	38 (25.3)
moderate	12 (8.0)
severe	65 (43.3)
Hemophilia B	35 (23.3)
mild	13 (8.7)
moderate	11 (7.3)
severe	11 (7.3)
Inhibitor history, N (%)	16 (11)
Any history of high titer inhibitor	5 (3)
Active inhibitor	5 (3)
Prophylaxis use, N (%)	
Receiving prophylaxis	90 (60)
Emicizumab	45 (30)
Gene therapy	3 (2)
Not receiving prophylaxis (on-demand treatment only)	54 (36)
On-demand treatment with pre-activity prophylaxis	6 (4)
HJHS with global gait (*N* = 115)	
Mean (SD)	28.3 (16.6)
Median	24
Minimum/maximum	4/73
HIV history, N (%)	9 (6)
HCV history, N (%)	37 (25)
Mental health history (N, %)	59 (39)
Depression	46 (31)
Anxiety	42 (28)
Substance abuse disorder	12 (8)
Other	5 (3)
Race (N, %)	
White	141 (94)
Black/African-American	1 (1)
Asian	2 (1)
American Indian or Alaska native	3 (2)
More than one race	2 (1)
Unknown	1 (1)
Ethnicity (N, %)	
Hispanic or Latino	14 (9)
Not Hispanic or Latino	132 (88)
Unknown	4 (3)

SD, standard deviation; HJHS, hemophilia joint health score; HIV, human immunodeficiency virus; HCV, hepatitis C virus

**Table 2:** Pain medication use profile for study participants

Pain treatment (*N* = 150)	Any reported use, *N* (%)	Scheduled Use ≥ 5 days per week, *N* (%)	Episodic Use < 5 days per week, *N* (%)
Pharmacological pain therapy	85 (57)	31 (21)	76 (51)
Opioids	17 (11)	9 (6)	12 (8)
Acetaminophen	56 (37)	4 (3)	52 (35)
NSAIDs (non-COX-2)	23 (15)	3 (2)	20 (13)
COX-2-NSAID	21 (14)	14 (9)	7 (5)
Gabapentin/pregabalin	4 (3)	4 (3)	0 (0)
SNRIs or SSRIs	3 (2)	3 (2)	0 (0)
Herbal supplements or homeopathic remedy	4 (3)	1 (1)	3 (2)
Cannabinoids	17 (11)	5 (3)	13 (9)
Multiple types	49 (33)	31 (21)	28 (19)
Non-pharmacological pain therapy	94 (63)	–	–
RICE	69 (46)	–	–
Physical therapy	14 (9)	–	–
Physical activity (exercise, swimming, yoga, etc.)	27 (18)	–	–
Mental health intervention	4 (3)	–	–
Other (massage, relaxation, distraction, acupuncture, etc.)	18 (12)	–	–
Alcohol	2 (1)	–	–
Multiple non-pharmacologic therapies	36 (24)	–	–

NSAIDs, nonsteroidal anti-inflammatory drugs; COX-2, cyclooxygenase-2; SNRIs, serotonin and norepinephrine reuptake inhibitors; SSRIs, selective serotonin reuptake inhibitors; RICE, rest/ice/compression/elevation

**Fig. 1:**
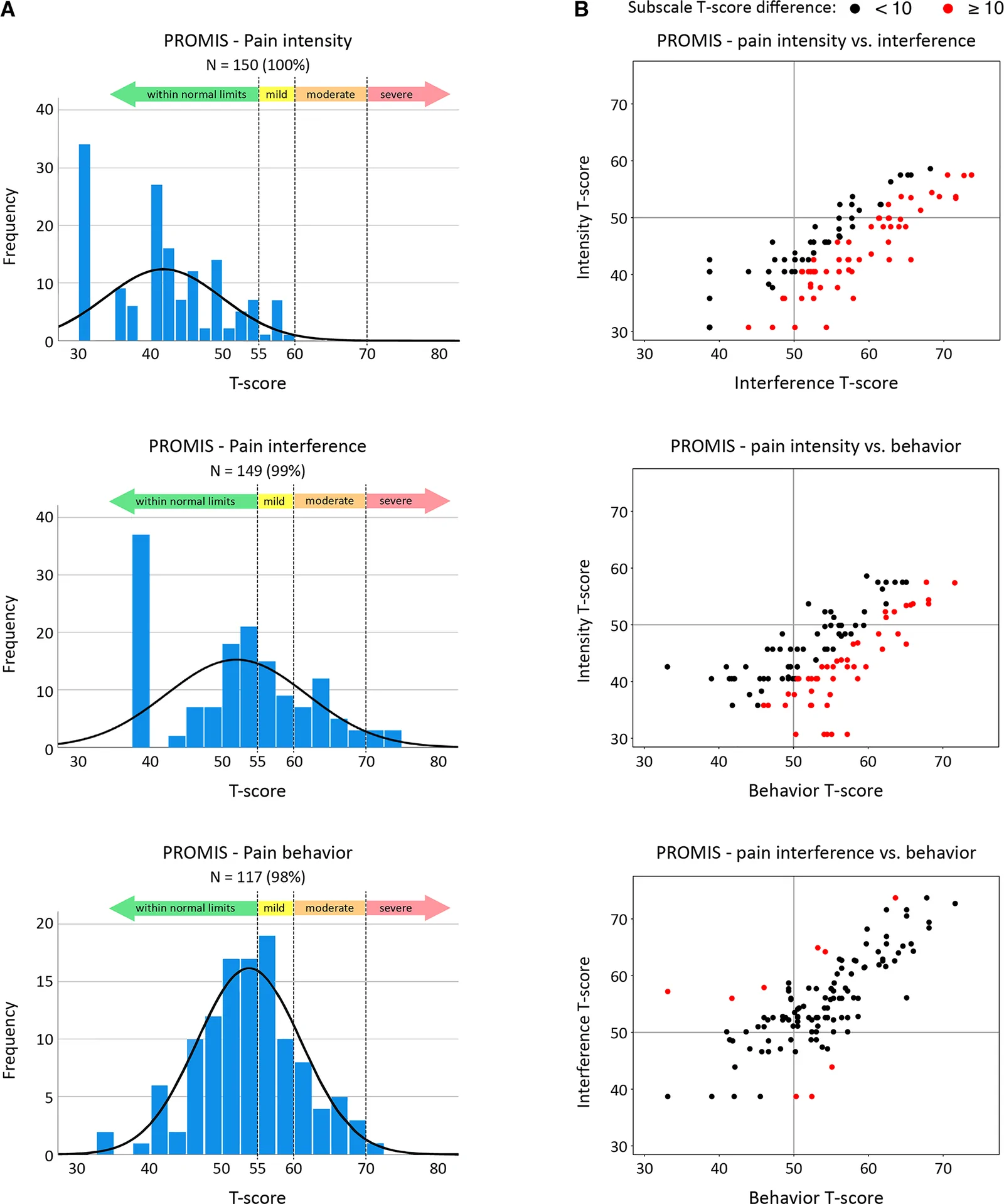
(**A**) T-score distributions from PROMIS pain intensity, pain interference, and pain behavior domains. The black curve indicates the normal distribution based on observed T-score frequencies. The number and percentage of participants responding to each domain item bank is indicated. Thirty participants indicated being pain-free when responding to the PROMIS pain behavior questionnaire and were not included in the pain behavior T-score analysis. PROMIS T-score ranges associated with different levels of the pain domain being measured were obtained from the PROMIS healthmeasures.net website [49] and are shown for added context. (**B**) Bivariate plots comparing PROMIS pain domain T-scores for study participants. Data points that represent “concordant scores” (T-score difference < 10 points) or “discordant scores” (T-score difference ≥ 10 points) for a study participant are indicated using black or red dots, respectively

**Fig. 2:**
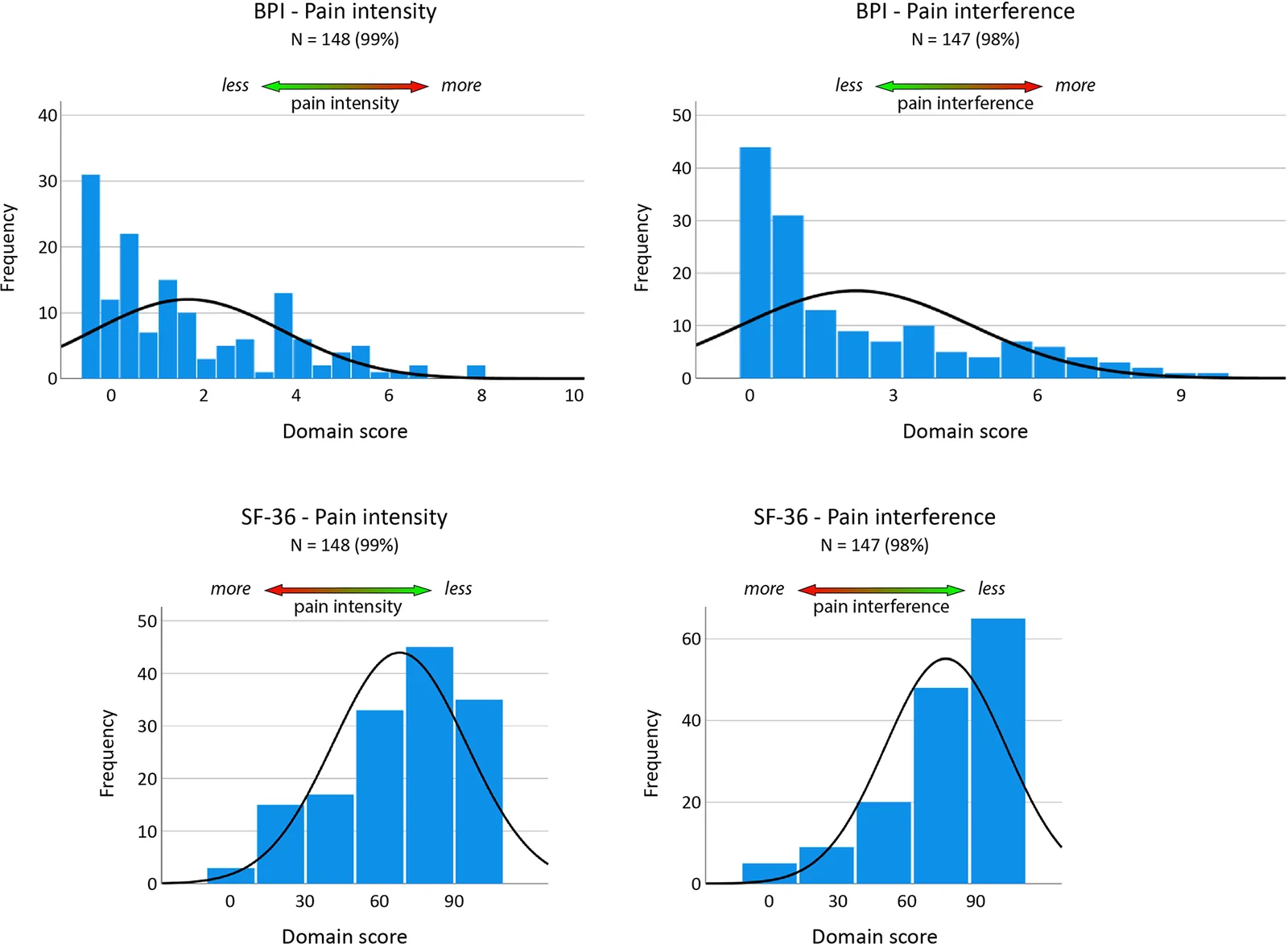
Distribution of scores from BPI and SF-36 pain intensity and pain interference domains. The black curve indicates the normal distribution based on observed domain score frequencies. The number and percentage of participants responding to each domain item bank is indicated

The distributions of T-scores for PROMIS pain intensity, pain interference, and pain behavior domains are shown in Fig. 1A, and pain domain scores for each participant are plotted and compared to one another in Fig. 1B. The mean (SD) T-score for the PROMIS intensity domain was 41.8 (8.1), while mean (SD) T-scores for the PROMIS interference and behavior domains were 52.0 (9.7) and 53.7 (7.2), respectively. Responses to the PROMIS pain intensity measure from 34/150 (23%) participants yielded T-scores of 30.7 (the lowest possible score), corresponding to a floor effect for respondents who reported no pain. Similarly, responses to the PROMIS pain interference CAT from 37/149 (25%) participants yielded T-scores of 38.7, indicating a floor effect for those participants whose daily life and activities are not impacted by pain. Comparable floor effects were indicated in the BPI pain intensity (31/150 participants, 21%) and pain interference domain scores (36/149 participants, 24%), and a larger floor effect was observed for the SF-36 pain interference scores (65/147 participants, 44%; Fig. 2). Thirty participants (20%) reported having no pain for all PROMIS pain behavior items administered and were excluded from PROMIS pain behavior domain T-score, validity, and test/retest reliability analyses. Participants completed the PROMIS pain intensity, interference, and behavior assessments in mean (SD) times of 1.0 (1.3), 1.2 (1.1), and 1.6 (1.7) minutes, respectively, and completed the BPI (11 items) and SF-36 bodily pain subscale (2 items) instruments in mean (SD) times of 1.5 (0.8) and 0.5 (0.6) minutes, respectively.

Correlations between PROMIS pain scores and the BPI and SF-36 bodily pain subscale scores were generally strong (Table 3). Internal consistency of the PROMIS pain intensity measure was demonstrated by Cronbach’s alpha of 0.86, and reliability of the PROMIS pain intensity, interference, and behavior CAT measures was also strong (marginal reliability 0.87, 0.91, and 0.88, respectively). The mean (median) number of PROMIS pain interference items that needed to be administered to achieve target precision during CAT-based assessment was 6 (4) with a range of 4–12 items, while the corresponding mean (median) number of PROMIS pain behavior items administered during CAT was 8 (4) with a range of 4–12 items. While PROMIS pain interference and behavior scores did not vary by hemophilia severity, differences in PROMIS pain intensity scores with increasing hemophilia severity reached statistical significance (*p* < 0.05; Table 4). Differences in BPI or SF-36 pain intensity scores with increasing hemophilia severity also either approached or achieved statistical significance (Table 4).

**Table 3:** Pearson’s correlation coefficients between the PROMIS and the BPI and SF-36 pain domains (convergent validity)

	PROMIS^†^		
	Intensity	Interference	Behavior
BPI			
Intensity	0.88 (147)	0.82 (147)	0.67 (116)
Interference	0.82 (146)	0.84 (146)	0.75 (115)
SF-36 bodily pain subscale^‡^			
Intensity	-0.88 (147)	-0.85 (147)	-0.66 (116)
Interference	-0.78 (146)	-0.84 (146)	-0.72 (115)

PROMIS, Patient-Reported Outcomes Measurement Information System; BPI, Brief Pain Inventory; SF-36, Medical Outcomes Study Short Form-36

^†^Pearson’s correlation coefficients are presented as r (sample size for the comparison). All correlations were statistically significant (*p* < 0.05)

^‡^The Pearson correlation coefficients between PROMIS and SF-36 pain domains are negative given the opposite orientation of the PROMIS and SF-36 pain domain scales (higher PROMIS T-scores reflect more of the pain construct being measured, while higher SF-36 pain scores reflect less pain)

**Table 4:** Differences in pain domain scores by hemophilia severity (known group validity)

Hemophilia severity comparison groups	Pain measure	Mean (SD) domain scores, *n*			*p*-value
mild vs. moderate vs. severe^†^		mild	moderate	severe	
	PROMIS				
	Intensity	39.8 (7.9), 51	40.8 (8.5), 23	43.4 (7.7), 76	**0.036**
	Interference	50.3 (10.1), 50	52.3 (11.1), 23	53.1 (9.0), 76	0.279
	Behavior	53.0 (7.4), 35	55.1 (7.8), 16	53.7 (7.0), 66	0.638
	BPI				
	Intensity	1.7 (2.2), 50	1.7 (1.8), 22	2.5 (2.0), 76	0.067
	Interference	1.8 (2.6), 49	2.2 (2.7), 22	2.5 (2.4), 76	0.289
	SF-36				
	Intensity	72.8 (27.9), 50	72.7 (24.3), 22	63.4 (26.4), 76	0.106
	Interference	81.5 (29.4), 50	73.9 (30.4), 22	75.0 (23.3), 75	0.341
non-severe (mild or moderate) vs. severe^‡^		non-severe		severe	
	PROMIS				
	Intensity	40.1 (11.6), 74		43.4 (7.7), 76	**0.012**
	Interference	50.9 (15.1), 73		53.1 (9.0), 76	0.171
	Behavior	53.7 (10.8), 51		53.7 (7.0), 66	0.957
	BPI				
	Intensity	1.7 (2.8), 72		2.5 (2.0), 76	**0.020**
	Interference	6.1 (3.8), 71		2.5 (2.4), 76	0.159
	SF-36				
	Intensity	72.8 (37.0), 72		63.4 (26.4), 76	**0.034**
	Interference	79.2 (42.2), 72		75.0 (23.3), 75	0.344

ANOVA and independent-samples t-test analysis results indicating whether PROMIS pain intensity, pain interference, and pain behavior domain scores differ significantly based on hemophilia severity group (known group validity). Corresponding results for BPI and SF-36 domain scores are also shown. Mean (SD) pain domain scores for each hemophilia severity group are indicated, as well as the sample size (n) for each comparison group. P-values that reached statistical significance (*p* < 0.05) are shown in bold. PROMIS, Patient-Reported Outcomes Measurement Information System; BPI, Brief Pain Inventory; SF-36, Medical Outcomes Study Short Form-36; ANOVA, analysis of variance; SD, standard deviation

^†^Results from ANOVA analysis

^‡^Results from independent-samples t-test analysis

Retest assessments were completed 24–72 h after the initial assessment by 104 study participants, with 73 of those participants reporting no change in pain. The repeat PROMIS pain assessments yielded ICCs of 0.85–0.95 (Table 5), suggesting acceptable test/retest reliability over 24–72 h. Participants (*n* = 23) who reported any decrease in pain (“a little better,” “moderately better,” or “much better”) on the GRC at the retest timepoint had decreased T-scores on the PROMIS pain intensity, interference, and behavior measures (mean decreases of 0.2, 1.3, and 0.7 points, respectively). Those (*n* = 8) who reported any increase in pain (“a little worse,” “moderately worse,” or “much worse”) on the GRC at the retest timepoint had increased T-scores on the PROMIS pain intensity, interference, and behavior measures (mean increases of 4.3, 3.6, and 5.2 points, respectively).

**Table 5:** ICCs obtained from repeat PROMIS pain measure evaluations 24–72 h after initial assessment (test/retest reliability)

PROMIS domain^†^	Number of test/retest assessments	ICC^‡^
Intensity	73	0.93
Interference	70	0.95
Behavior	46	0.85

PROMIS, Patient-Reported Outcomes Measurement Information System; ICCs, intraclass correlation coefficients

^**†**^Of the 73 participants who reported no change in pain 24–72 h after the initial assessment, 3 completed only the pain intensity questionnaire on retest and another 24 either did not complete the pain behavior questionnaire (1 participant) or reported being pain-free during the initial test and/or retest evaluation (23 participants)

^**‡**^All correlations were statistically significant (*p* < 0.05)

Ninety-nine of 150 study participants agreed to be contacted after the initial study visit to schedule a time for content validity interviews. Attempts to contact participants were made for 18 participants, with 10 successful contacts with interviews scheduled. Eight participants who completed questionnaires (5%) were interviewed for content validity testing, with all interviews being conducted in English. Further attempts to conduct interviews were not completed due to the onset of the COVID-19 pandemic in March 2020. Participants typically indicated that all of the items presented in the PROMIS pain interference questionnaire were relevant to their pain experience, and no interview participant identified any aspects of pain interference relevant to their experiences that were not covered by the items in the PROMIS pain interference item bank. After reviewing each item in the pain interference item bank, three of the eight participants (38%) reported ambiguity in the question “How often was pain distressing to you?” (PAININ24), with the word “distressing” being noted as vague or non-specific by all three participants.

## Discussion

Pain assessment plays a crucial role in hemophilia care, as the interpretation of pain (either by the patient, caregiver, or clinician) drives treatment and management decisions. Legacy PRO assessment tools used in hemophilia research vary widely in scoring methodologies, respondent burden, and psychometric properties that complicate comparison of study results, and the lack of full psychometric characterization for these instruments represents a barrier to routine use in clinical settings [31, 32]. Standardized PRO measures that reliably and systematically assess hemophilia-related pain would be of great value, allowing patients and clinicians to consistently identify and treat bleeding episodes, modify treatment for pain, and assess responses to interventions.

Items included in adult or pediatric PROMIS measures are designed to be insensitive to the effects of respondent age, sex, and education level, and allow comparison of outcomes across age ranges within the respective adult or pediatric populations and from other disease settings [10]. PROMIS measures have been shown to function well in many chronic disease populations [33–36], including hemophilia [15–17, 37, 38], von Willebrand disease, and other rare bleeding disorders [39, 40]. The PROMIS pain interference domain has been shown to adequately assess outcomes in adult and pediatric PwH [15–17], and the single-item pain intensity measure included in the PROMIS Profile-29 could discern mild and severe hemophilia forms in adult PwH [15].

Our current study examined the reliability and validity of PROMIS pain intensity, pain interference, and pain behavior measures in 150 adult PwH. The extent of joint disease and the observed prevalence of mental health problems in study participants (Table 1) reflects an unbiased sampling of male PwH of diverse age, disease severity, inhibitor status, joint health, and pain experience being treated at the University of Colorado HTC. The PROMIS pain measures’ scores showed good internal consistency, as well as adequate test/retest reliability over 24–72 h. In addition, participants who reported change (either better or worse) at this time point showed, on average, corresponding changes in the PROMIS pain measures, though the number of participants in these groups was too small for meaningful statistical testing or comparisons. Strong content validity of the PROMIS pain interference measure was also supported, with no coverage gaps revealed during cognitive interviews. One item related to pain-related distress from the pain interference item bank was characterized by 3/8 participants as being ambiguous and therefore may warrant careful consideration before inclusion into customized short form questionnaires. The high percentages of participants who completed the PROMIS pain domain assessments (98%-100%), as well as the relatively short time taken to complete each PROMIS pain assessment (mean of 1.0–1.6 min) indicates that the administration of these measures is potentially feasible in the outpatient clinic setting.

The mean PROMIS pain intensity score (41.8) was 10–12 points lower than the mean PROMIS pain interference and behavior scores (52.0 and 53.7, respectively). Conceivably, the lower mean pain intensity score may reflect a response shift phenomenon [41] for participants who have endured episodes of severe pain in the past and have adjusted to those experiences, adapting to elevated baseline pain levels and thereby reporting milder pain intensity during the PROMIS assessments. Alternatively, lower pain intensity ratings might reflect actual lower levels of pain intensity resulting from recent use of a pain medication, or from avoidance of activities that lead to increased pain. In this later instance, higher mean pain interference and behavior scores may suggest that the impact of pain on the daily activities of these patients is greater than what might be inferred from the pain intensity score alone, and highlights the importance of performing a comprehensive assessment of multiple pain domains to avoid underestimating the true burden of pain in PwH. The bivariate comparisons of the PROMIS domains shown in Fig. 1B illuminate the small number of individuals who have “discordant” responses among the three domains. Reviewing these results with individuals during clinic visits often revealed new insights related to the participants’ experiences with pain, reinforcing the notion that clinical use of the PROMIS measures of pain is greatly enhanced by reviewing results with patients in real-time. Exploring the relationships between pain behavior, pain intensity, and pain interference is potentially useful in understanding the impact of chronic pain in PwH who suffer from debilitating joint disease, which can strike PwH at an early age and become a major factor in daily behaviors, choices, and life aspirations. Discussion of PROMIS pain assessment results between patient and provider can potentially lead to a better understanding of hemophilia-related pain for the patient and allow providers to tailor more effective pain management programs for PwH.

The reasons why measures of pain intensity could distinguish among different hemophilia severities while pain interference and behavior measures did not (Table 4) are unclear. Pain intensity is conceivably more reflective of underlying joint damage, which is expected to be more significant with increasing hemophilia severity [42], while pain interference and behavior depend largely on other characteristics not tied to hemophilia severity, such as avoidance of activities with increased risk of injury or bleeding. Individuals with severe hemophilia who are using prophylaxis (as was the case with most individuals with severe hemophilia in our study) may be less likely to alter their activities as compared to those who are not, given that prophylaxis reduces the risk of bleeding. Such behavioral differences could potentially impact either pain interference or pain behaviors in ways that reduce or eliminate differences between known hemophilia severity groups. These findings underscore a growing appreciation that baseline factor activity levels do not necessarily correlate with bleeding phenotype or a person’s experience with pain related to hemophilia [38, 43, 44].

While we noted floor effects for PROMIS pain intensity and interference domains (more than 15% of participants with the lowest possible scores), as well as for legacy instrument measures (BPI pain intensity and interference domains, and the SF-36 pain interference domain), such observations are not unexpected given that multiple participants included in the study were not experiencing pain when they completed the questionnaires. While floor effects reduce the utility of patient-reported measures in some circumstances, measuring pain for clinical purposes may not be hampered by such floor effects, as those patients with low scores likely need no intervention for pain. PROMIS pain domain floor effects have also been observed in prior studies where a substantial proportion of PwH were using prophylaxis [15, 16]. While not utilized for this study, PROMIS measures with built-in “screen-to-CAT” functionality are available that stop the test for those who report no pain upon initial questioning, reducing respondent burden [20, 21]. Further studies in PwH having reduced access to effective prophylactic treatments may be needed to better characterize whether clinically relevant ceiling effects in these PROMIS pain measures exist in those populations expected to have higher levels of pain.

**Study limitations** The generalizability of our study’s results may be limited by our study sample, which was drawn from a single hemophilia treatment center and consisted of participants who were largely (88%) white, non-Hispanic adult males. Hemophilia has no particular racial or ethnic predisposition, and the demographics of our study sample reflect the demographics of this particular clinic and local population. In addition, women, girls, and people with the potential to menstruate can also be affected by hemophilia and pain and were not included in this study. A further limitation is that we did not collect responses from health measures questionnaires that were unrelated to pain, and so the discriminant validity of the PROMIS pain measures could not be assessed. Additionally, our convergent validity analysis that characterized correlations between the PROMIS and SF-36 pain domains relied on a single SF-36 pain domain item score, rather than a pain subscale score based on multiple items. We therefore surmise that these observed correlations are likely attenuated due to the increased measurement error and decreased score variability that ensue when using a single item instead of a multi-item scale, and that these particular correlation coefficients between the PROMIS and SF-36 pain domain scores represent conservative estimates of convergent validity. Also, while we did not directly assess measurement error in our study, our test-retest reliability results indicate that the PROMIS pain instruments are stable over 24–72 h, a finding that is consistent with an acceptable level of measurement error. Though our study did not explore the structural validity of the PROMIS pain questionnaires, these measures have been previously tested to ensure that only a single construct is assessed with each instrument [11, 22, 45].

While PROMIS pain quality measures were not evaluated in our study sample, distinguishing between nociceptive and neuropathic pain in PwH is generally not clinically useful given that most PwH have joint pain as the primary pain manifestation. Administration of the PROMIS pain quality questionnaire was therefore not included in the study design to avoid undue respondent burden. Another limitation of the study was the relatively small number of qualitative interviews that were conducted for evaluating content validity. The timing of the study overlapped with the COVID-19 pandemic, which substantially impacted the logistics of conducting these interviews. PROMIS item banks were developed using robust and well-characterized methods, and the measures demonstrate content validity in multiple populations [12, 46–48]. Given the lack of content validity testing reported for this particular population, we feel that reporting our results is worthwhile, noting the potential limitations of a small sample size.

## Conclusions

Results from this study support PROMIS pain intensity, pain interference, and pain behavior measures as valid and reliable measures and potential alternatives to legacy instruments when assessing pain in adult PwH. The number of PROMIS items presented as well as the short administration times observed (comparable to BPI and SF-36 administration times) are consistent with a low respondent burden. Further characterization from longitudinal studies is needed to establish PROMIS pain score utility over time and to assess responsiveness of the measures to changes in pain over time. The study outcomes reported here expand on the limited available data regarding pain assessment using PROMIS in PwH, and can ultimately support the use of PROMIS pain measures in clinical practice for hemophilia.

## Supplementary Information

Below is the link to the electronic supplementary material.

Supplementary Material 1

## Data Availability

The datasets used and/or analyzed during the current study are available from the corresponding author on reasonable request.

## Abbreviations

ANOVA: Analysis of variance
BPI: Brief Pain Inventory
CAT: Computerized adaptive testing
COX-2: Cyclooxygenase-2
GRC: Global rating of change
HCV: Hepatitis C virus
HIV: Human immunodeficiency virus
HJHS: Hemophilia joint health score
HTC: Hemophilia and Thrombosis Center
ICC: Intraclass correlation coefficient
IRT: Item response theory
NIH: National Institutes of Health
NSAID: Nonsteroidal anti-inflammatory drug
PRO: Patient-reported outcome
PROMIS®: Patient-Reported Outcomes Measurement Information System^®^
PwH: Persons with hemophilia
REDCap: Research Electronic Data Capture
RICE: Rest/ice/compression/elevation
SD: Standard deviation
SE: Standard error
SF-36: Medical Outcomes Study Short Form-36
SNRI: Seratonin and norepinephrine reuptake inhibitor
SSRI: Selective seratonin reuptake inhibitor
Description of data: Characteristics of PRO instruments used in the current study
