# Supplementary material for: Reliability and validity of patient-reported outcomes measurement information system® (PROMIS®) measures of pain in adult males with hemophilia
Source: J Patient Rep Outcomes. 2026 Aug 19;10:212. doi: 10.1186/s41687-026-01176-0 (PMC13759871; doi:10.1186/s41687-026-01176-0)
Supplement: Supplementary file 1 [file 41687_2026_1176_MOESM1_ESM.docx]

**Supplementary Information**

**Supplementary Table 1:** Characteristics of PRO instruments used in the current study.

| **Instrument** | **Domains measured** | **Number of items**  **presented** | **Mode of administration** |
| --- | --- | --- | --- |
| PROMIS | Pain intensity  Pain interference  Pain behavior | 3  4-12^†^  4-12^†^ | Static form  CAT  CAT |
| BPI | Pain intensity  Pain interference | 11 | Static form |
| SF-36 bodily pain subscale | Pain intensity  Pain interference | 2 | Static form |

PRO, patient-reported outcome; PROMIS, Patient-Reported Outcomes Measurement Information System; CAT, computerized adaptive testing; BPI, Brief Pain Inventory; SF-36, Medical Outcomes Study Short Form-36.

^†^CAT allows specification of the minimum and maximum number of items presented to study participants.
